# Supplementary material for: Glutathione Non-Covalent Binding Sites on Hemoglobin and Major Glutathionylation Target betaCys93 Are Conservative among Both Hypoxia-Sensitive and Hypoxia-Tolerant Mammal Species
Source: Int J Mol Sci. 2023 Dec 19;25(1):53. doi: 10.3390/ijms25010053 (PMC10778717; doi:10.3390/ijms25010053)

Supplement

Table S1. List of hemoglobins of hypoxia tolerant and sensitive species.

Species	alpha	Cys	His	Arg	Lys	beta	Cys	His	Arg	Lys	gamma	Cys	His	Arg	Lys	delta	Cys	His	Arg	Lys	epsilon	Cys	His	Arg	Lys	theta	Cys	His	Arg	Lys	zeta	Cys	His	Arg	Lys	mu	Cys	His	Arg	Lys
Homo sapiens (Human)	P69905	1	10	3	11	P68871	2	9	3	11	P69892	1	7	3	12	P02042	2	7	4	11	P02100	1	7	2	14	P09105	2	8	8	5	P02008	1	7	6	9	Q6B0K9	2	7	6	3
Theropithecus gelada (Gelada baboon)	P01932	1	11	3	14	A0A8D2FZZ9	2	9	2	12						A0A8D2FZR4	3	6	3	13	A0A8D2FYY1	1	7	3	13	A0A8D2EQL5	2	7	9	5						A0A8D2GNE5	2	8	6	3
						P02029	2	9	2	12						A0A8D2K6V2	2	10	3	11	A0A8D2G0Y9	4	4	4	10															
																					A0A8D2G2K3	2	5	2	12															
Rhinopithecus roxellana (Golden snub-nosed monkey) (Pygathrix roxellana)	A0A2K6R518	1	10	3	11	A0A2K6Q8D1	2	9	3	11	A0A2K6RPT9	1	6	4	11	A0A2K6P3G0	2	8	3	11	A0A2K6R3K8	1	7	2	14	A0A2K6NMM6	2	7	9	5	A0A2K6PNW2	1	6	6	9	A0A2K6RPM9	2	8	6	3
																A0A2K6P3I2	3	5	3	13											A0A2K6Q4K7	2	6	6	9					
Colobus guereza (Mantled guereza) (Eastern black-and-white colobus monkey)	A0A3G2VT56	1	11	3	11	Q9TT33	2	7	6	9																														
Ailurus fulgens (Lesser panda) (Red panda)	P18969	2	10	3	11	P18982	2	8	2	14																														
Ursus thibetanus (Asiatic black bear) (Selenarctos thibetanus)	P68236	1	10	3	11	P68012	2	8	2	14																														
Lama glama (Llama)	P01973	1	10	3	13	P68226	1	8	6	11																														
Lama guanicoe (Guanaco) (Lama glama guanicoe)	P67815	1	11	3	13	P68229	1	8	6	11																														
Vicugna pacos (Alpaca) (Lama pacos)	P67816	1	11	3	13	P68228	1	8	6	11											A0A6I9I625	1	6	4	12	A0A6I9I5T0	1	8	10	5	A0A6I9IUX8	2	7	6	9	A0A6I9I7A0	2	8	7	4
	A0A6I9IV03	1	11	3	10																A0A6I9I7L9	1	9	4	11											A0A6I9I8P6	2	7	8	2
																					A0A6I9I4T5	1	8	5	10															
Lama vicugna (Vicugna) (Vicugna vicugna)	P07425	1	11	3	13	P68227	1	8	6	11																														
Pantholops hodgsonii (Chiru) (Tibetan antelope)	Q0ZA50	1	9	3	11	F1DSD0	1	8	5	11	J7LGZ1	1	6	6	10																									
Capra hircus cretica	A0A7D5Y174	1	9	3	11	A0A7D6C7I6	1	9	5	11																														
						A0A7D6C9N7	1	9	5	11																														
Capra hircus (Goat)	P0CH25	1	9	3	11	P02077	1	9	4	12	P02082	1	6	6	10						A0A452F723	2	8	5	12	A0A452E971	2	9	9	5	A0A452EFQ5	2	7	6	8	A0A452DRK3	0	8	8	3
	P0CH26	1	10	3	11	A0A452FWW3	1	5	8	9											P02102	1	7	4	12	A0A8C2R4C8	1	7	6	3	P13786	2	7	6	8	A0A8C2R3G3	1	7	6	3
						A0A452FX49	1	8	5	11											P02105	2	8	4	11						A0A8C2R2Z3	1	5	12	6					
						A0A452FXQ3	1	8	5	11																														
						P02078	1	8	5	11																														
Capra caucasica caucasica	A0A7D5Y015	1	9	3	11	A0A7D6C535	1	8	5	11																														
						A0A7D6BXX8	1	9	5	11																														
Capra nubiana (Nubian ibex) (Capra ibex nubiana)	A0A7D5Y1A3	1	9	3	11	A0A7D6C4X7	1	8	5	11																														
						A0A7D6C2F8	1	9	4	12																														
Bos mutus grunniens (Wild yak) (Bos grunniens)	P01968	0	10	3	11	P02072	1	7	4	13																A0A8B9XIZ0	2	9	6	6	A0A8B9XKA1	2	7	7	7	A0A8B9XKR7	1	7	8	3
	P01967	0	9	3	11	D4QBF3	1	6	4	13																					A0A8B9XRJ2	2	7	7	8					
						D4QBF4	1	7	4	13																														
Bos taurus (Bovine)	P01966	0	10	3	11	P02070	1	6	4	13											P06643	1	8	4	10						F1MMI1	2	7	7	7	A1A4Q3	1	7	8	3
						P02081	2	5	6	11											P06642	2	7	4	10						E1BAP8	2	7	8	7					
																					E1B976	2	7	4	14															
																					E1BIF9	1	7	4	12															
Cavia porcellus (Guinea pig)	P01947	1	10	3	11	P02095	2	11	3	11																H0WAL2	3	6	3	8										
	A0A222AH49	1	10	2	10	A0A7T7JJ63	2	11	3	11																														
Sus scrofa (Pig)	P01965	1	11	3	11	P02067	1	8	5	11											P02101	1	7	3	12	P04246	1	7	3	12	P02009	1	6	5	9	F1RGX5	1	8	8	3
	A0A4X1UIB6	1	11	3	11																					K7GNR8	2	7	11	7	F1RGX7	1	6	5	9	A0A5G2QP95	2	7	11	7
	A0A4X1UM57	1	11	3	11																					A0A4X1UMQ7	2	7	11	7	A0A8D1WEI1	2	3	5	7					
	A0A4X1UMN0	1	11	3	11																					A0A8D1WDA8	2	7	11	7	A0A4X1UI72	1	6	5	9					
	A0A4X1UMN5	1	11	3	11																																			
	A0A4X1UMP8	2	10	3	11																																			

Species	alpha	Cys	His	Arg	Lys	beta	Cys	His	Arg	Lys	gamma	Cys	His	Arg	Lys	delta	Cys	His	Arg	Lys	epsilon	Cys	His	Arg	Lys	theta	Cys	His	Arg	Lys	zeta	Cys	His	Arg	Lys	mu	Cys	His	Arg	Lys
Hydrodamalis gigas (Steller's sea cow)	A0A481WNT7	1	9	5	7	A0A481WPM8	2	10	4	9	A0A481WPK4	1	4	3	13						A0A481WND8	1	5	3	10						A0A481WNG0	1	6	6	8	A0A481WQ33	2	9	6	3
	A0A481WNB4	1	10	2	11	A0A481WR13	2	10	4	10	A0A481WQZ5	1	4	4	14						A0A481WNH0	1	7	3	13															
Dugong dugon (Dugong) (Trichechus dugon)	A0A481WNF4	1	10	5	10	A0A481WQB0	2	10	3	12	Q45XH5	1	4	4	14	Q45XI7	2	10	3	12	A0A481WND9	1	7	3	13						A0A481WNH8	1	6	6	9	A0A481WNV4	3	11	6	3
											A0A481WQ84	1	4	4	14																									
											A0A481WQ07	1	4	4	14																									
Trichechus manatus latirostris (Florida manatee)	A0A2Y9ECS7	1	9	5	7	A0A2Y9ECT2	2	8	4	12	A0A2Y9E9M2	1	4	5	14						A0A2Y9E8B4	1	7	3	13						A0A2Y9DEZ0	1	6	6	10	A0A2Y9DFF6	2	10	6	3
Marmota flaviventris (Yellow-bellied marmot)	T1WIE1	1	11	3	10	T1WI68	1	9	2	13																														
	T1WIZ1	1	11	3	10	T1WJ01	1	9	2	13																														
						T1WIZ6	1	9	2	13																														
						T1WIF1	1	9	2	13																														
						T1WHQ1	1	9	2	13																														
						T1WI63	1	9	2	13																														
Chinchilla lanigera (Long-tailed chinchilla) (Chinchilla villidera)	A0A8C2W7L6	1	11	3	11	A0A8C2W0C9	1	11	3	10											A0A8C2W8L3	1	7	3	14	A0A8C2W3W5	2	6	6	7	A0A8C2W2V0	1	7	5	9					
																					A0A8C2W3S0	1	7	3	13															
Ctenomys rionegrensis	A0A222AH35	1	11	2	10	A0A7T7FF42	1	11	3	10																														
	A0A222AH43	1	11	2	10																																			
Heterocephalus glaber (Naked mole rat)	G5BXY2	2	5	7	9	A0A0A7HRP5	1	13	3	10	G5BS34	1	6	3	14						G5BS35					G5BXY0	1	6	5	8	A0A0P6IYM2	1	7	5	8					
	G5BXY4	6	4	5	7	G5BS33	1	12	3	10																					G5BXY5	1	5	5	7					
	G5BXY1	1	11	3	10	G5BYJ8	1	13	4	10																					G5CB64	1	13	4	10					
																															G5BXY3	1	7	5	8					
Ochotona curzoniae (Black-lipped pika)	A4GTS5	1	10	3	11	Q0PG38	1	10	2	12																														
	F6MFG2	1	9	3	11																																			
Procavia capensis habessinica (Abyssinian hyrax)	P01957	1	10	4	11	P02086	1	11	4	10																														
Spalacopus cyanus (Coruro)	A0A222AH41	1	11	2	10	A0A7T7FF05	1	11	3	10																														
	A0A222AH48	1	11	2	10																																			
Spalax ehrenbergi (Middle East blind mole rat) (Nannospalax ehrenbergi)	P01943	1	12	4	10	P02090	1	9	2	13																														
Nannospalax galili (Northern Israeli blind subterranean mole rat) (Spalax galili)	A0A8C6R454	1	12	4	10	A0A8C6REJ7	1	9	2	13											A0A8C6RZS6	1	6	3	13	A0A8C6W833	2	9	5	8	A0A8C6R034	1	5	6	9					
						A0A8C6S226	1	9	2	13																														
						A0A8C6RUI9	1	4	8	13																														
						A0A8C6RII4	4	7	2	13																														
Talpa europaea (European mole)	P01951	2	11	3	11	P02061	2	6	4	13																														
Tamias umbrinus (Uinta chipmunk) (Neotamias umbrinus)	T1YT26	3	11	2	10	T1YUG6	1	7	2	11																														
	T1YVM3	3	11	2	10																																			
Erinaceus europaeus (Western European hedgehog)	P01949	1	10	3	11	A0A1S3WPY1	2	6	4	13											A0A1S3ADV4	1	7	3	13	A0A1S3A241					A0A1S3A1U8	2	8	6	9	A0A1S3A1W3	3	7	6	2
						P02059	2	6	4	13																					A0A1S3A1V5	1	6	8	7					
						A0A1S3ADW1	1	5	3	12																														
						A0A1S3AE42	2	6	4	13																														

Species	alpha	Cys	His	Arg	Lys	beta	Cys	His	Arg	Lys	gamma	Cys	His	Arg	Lys	delta	Cys	His	Arg	Lys	epsilon	Cys	His	Arg	Lys	theta	Cys	His	Arg	Lys	zeta	Cys	His	Arg	Lys	mu	Cys	His	Arg	Lys
Rattus norvegicus (Rat)	A0A0A0MP82					P02091	2	10	3	12																					A6HDA9	1	9	5	9					
	A0A1K0FUB4	3	10	3	12	A0A0G2JSW3	1	10	3	12																					G3V8R3	1	9	5	9					
	A0A8I5ZYH2	2	10	2	12	A0A0G2JTW9	2	10	3	12																														
	A0A8I6AI13	1	11	5	8	A0A8I5ZXF1	2	10	2	12																														
	A0A8I6AWV0	1	9	2	12	A0A8I6AUL4	1	9	2	12																														
	A0A8I6GMB4	2	11	4	9	A0A8L2R1Q6	2	10	3	12																														
	A0A8L2QLW7	2	11	4	9	A0A8L2R6L7	2	9	2	12																														
	P01946	3	10	3	12	A0A8I6ASA5	1	11	5	8																														
						P11517	2	10	3	12																														
Mus musculus (Mouse)	P01942	1	11	3	11	B1Q450	1	9	3	11											P02104	1	6	3	12						P06467	2	7	6	9					
						P02088	2	9	3	11																					Q78PA4	2	7	6	9					
	A7M7S6	2	8	12	10	P02089	2	8	3	12																														
						P04443	1	7	3	14																														
						P04444	1	8	4	13																														
						E9Q223	1	7	4	12																														

Figure S1. Phylogenetic reconstruction of hemoglobin protein sequences by similarity using BLOSUM62 matrix UPGMA clustering.

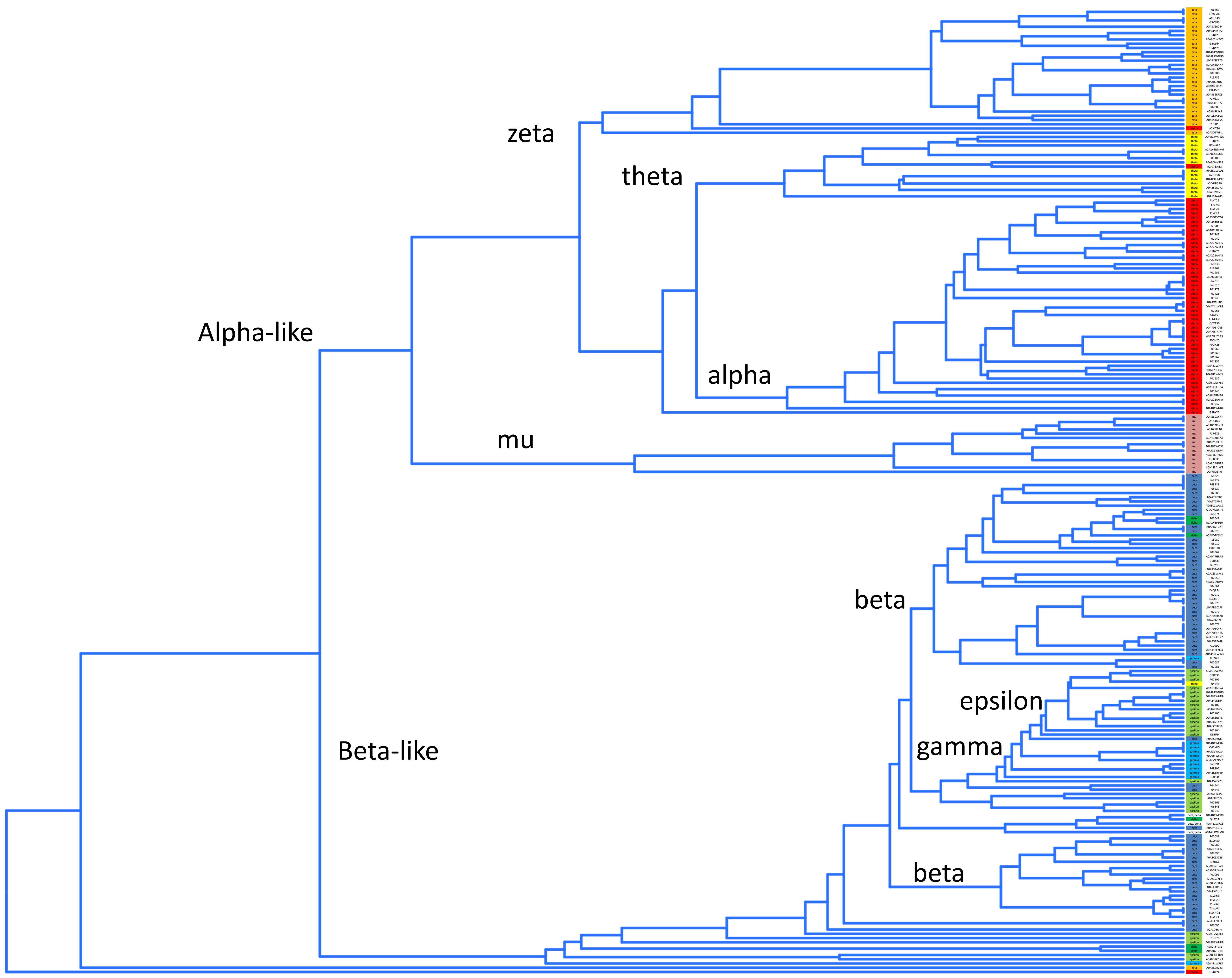

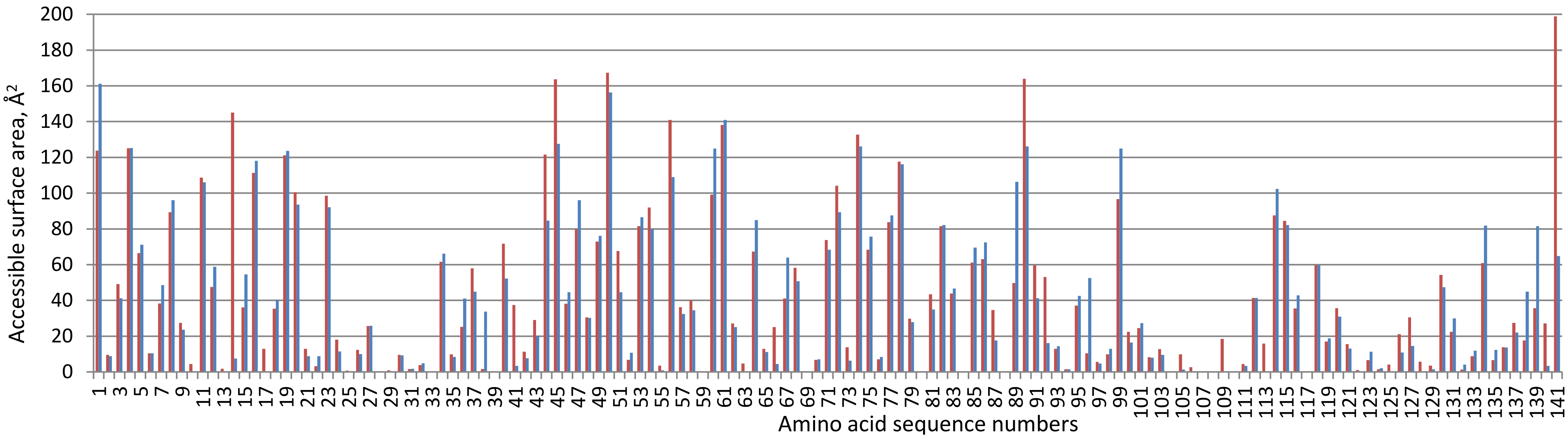

Figure S2. Accessible surface area of amino acid residues of the alpha-subunit of human hemoglobin in the oxy-form (based on the oxy 1r1x(red) and deoxy 1a3n(blue) structures).

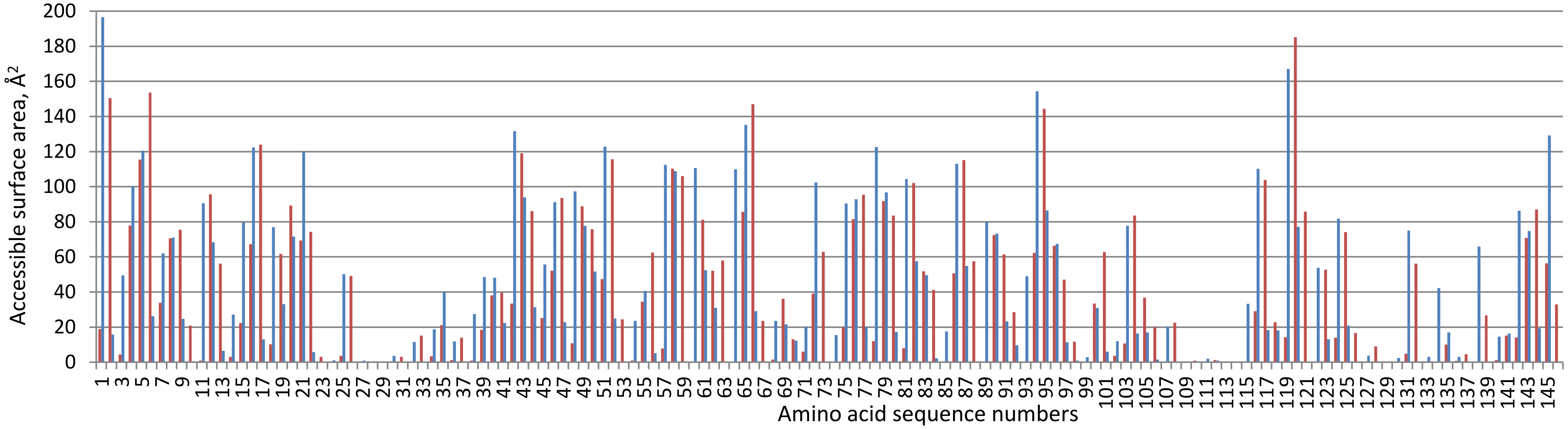

Figure S3. Accessible surface area of amino acid residues of the beta-subunit of human hemoglobin in the oxy-form (based on the oxy 1r1x(red) and deoxy 1a3n(blue) structures).

Figure S4. Sequence alignment of hemoglobin alpha-subunit both hypoxia-tolerant and hypoxia-sensitive species.

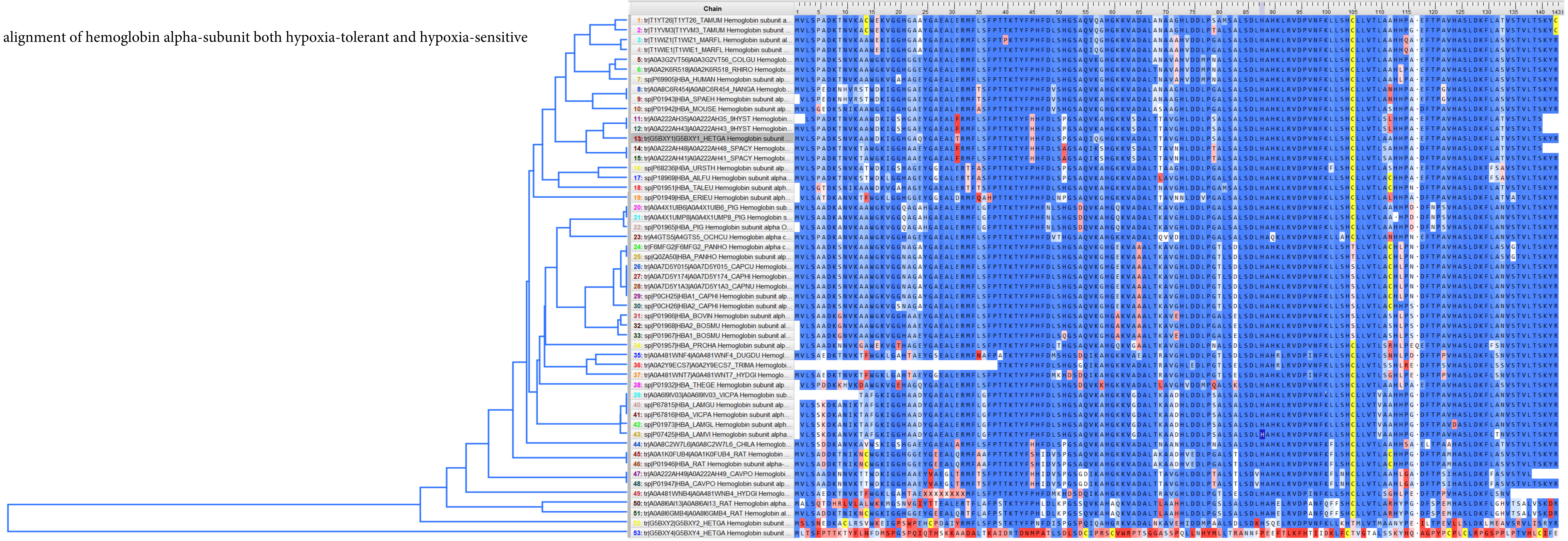

Figure S5. Sequence alignment of hemoglobin beta-subunit both hypoxia-tolerant and hypoxia-sensitive species.

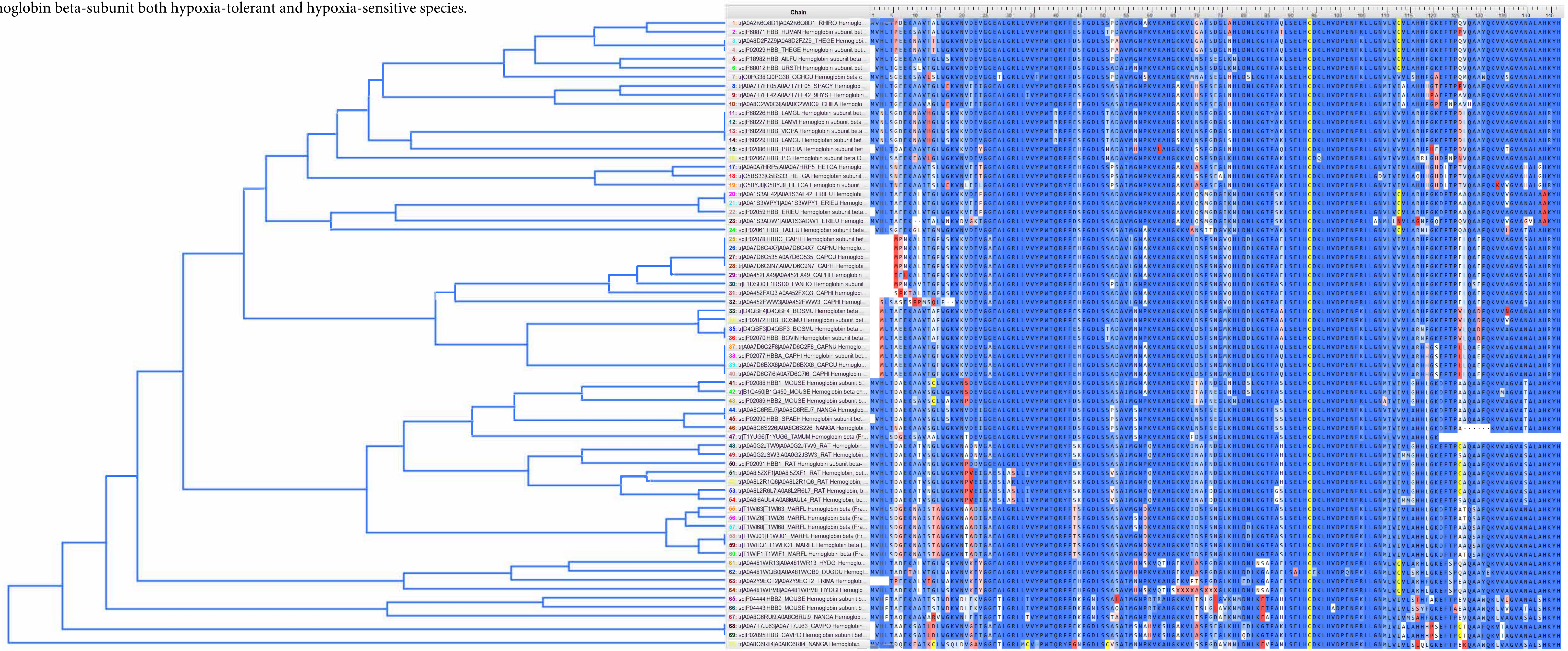

Supplement: Supplementary file 1 [file ijms-25-00053-s001.zip › ijms-2759090-supplementary.pdf]
